# Utility of Adjunctive Impella Support to Venoarterial Extracorporeal Membrane Oxygenation for a Refractory Electrical Storm

**DOI:** 10.7759/cureus.64382

**Published:** 2024-07-12

**Authors:** Yusuke Akazawa, Haruhiko Higashi, Toru Miyoshi, Shinji Inaba, Osamu Yamaguchi

**Affiliations:** 1 Department of Cardiology, Pulmonology, Hypertension and Nephrology, Ehime University, Toon, JPN; 2 Department of Cardiology, Pulmonology, Hypertension and Nephrology, Ehime University Graduate School of Medicine, Toon, JPN

**Keywords:** intra-aortic balloon pump, iabp, impella cp, ventricular fibrillation (vf) storm, cardiogenic shock, venoarterial extracorporeal membrane oxygenation

## Abstract

Venoarterial extracorporeal membrane oxygenation (VA-ECMO) stabilizes hemodynamics in an electrical storm leading to cardiogenic shock. However, adverse effects of VA-ECMO are increased left ventricular (LV) afterload and LV end-diastolic pressure due to retrograde blood return. These adverse effects could be ameliorated by LV unloading with Impella insertion. This case illustrates the possible efficacy of adjunctive Impella insertion for a refractory electrical storm that is resistant to defibrillation under mechanical support with VA-ECMO for cardiogenic shock.

## Introduction

Ventricular fibrillation (VF) is the most serious concern secondary to coronary artery occlusion despite successful revascularization and can develop into an electrical storm leading to cardiogenic shock [1]. To date, however, the management of electrical storm refractory to antiarrhythmic drugs is challenging, especially under cardiogenic shock. Although it has been reported that mechanical circulatory support (i.e. venoarterial extracorporeal membrane oxygenation (VA-ECMO), Impella, or left ventricular assist device) could be useful for the termination of an electrical storm, it remains in debate [1-3]. Therefore, the accumulation of effective therapeutic strategies for its resolution under cardiogenic shock is mandatory.

## Case presentation

**Figure 1:**
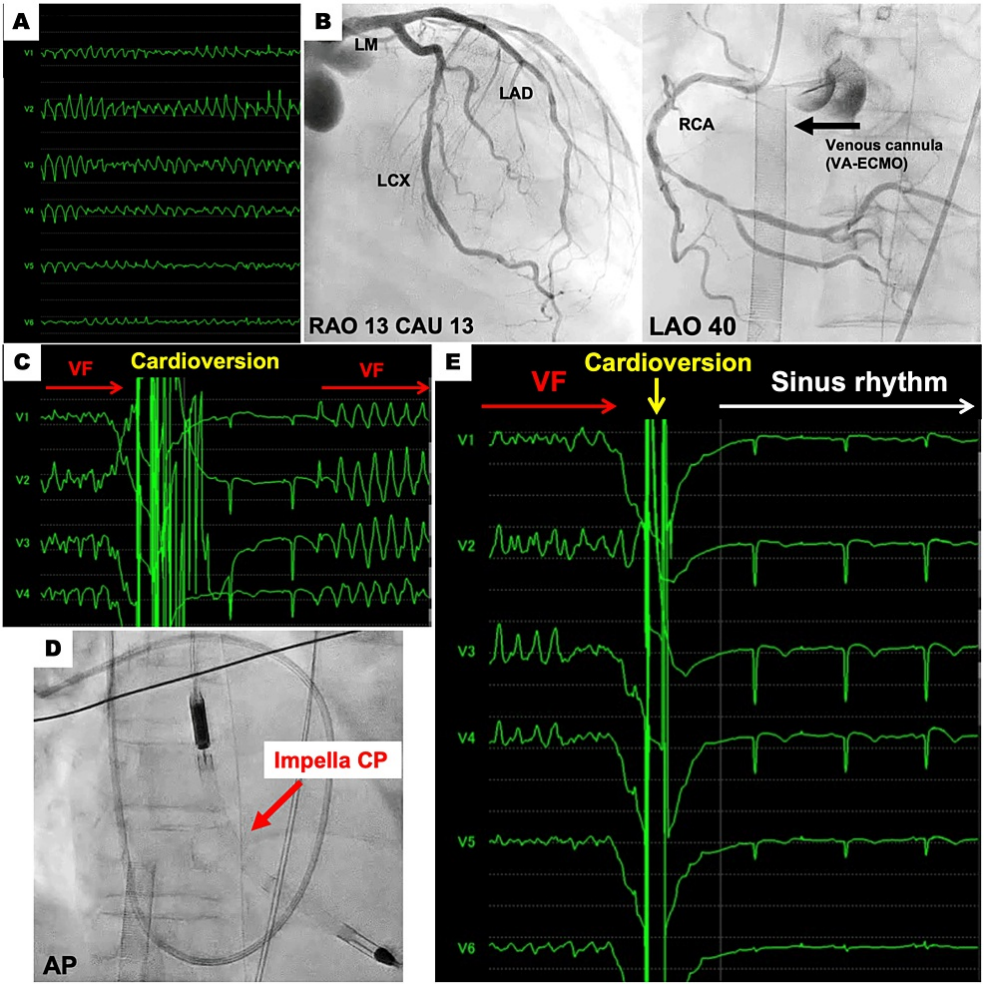
Monitor electrocardiogram (ECG) and emergent coronary angiography (A) Monitor ECG showing ventricular fibrillation (VF), (B) Coronary angiogram showing no obstruction, (C) Monitor ECG showing VF recurrence soon after direct-current cardioversion, (D) Fluoroscopic image showing the inserted Impella CP, (E) Monitor ECG showing sinus rhythm recovery with defibrillation after adjunctive Impella insertion

A 69-year-old man was transferred to our hospital for managing cardiogenic shock following an electrical storm (Figure 1).

The patient was rushed to the previous hospital due to worsening dyspnea. He was diagnosed with ischemic heart failure due to subacute myocardial infarction and was undergoing heart failure control with the following medications as the daily dose: aspirin 100 mg, prasugrel 3.75 mg, bisoprolol 4 mg, azosemide 30 mg, spironolactone 25 mg, atorvastatin 10 mg, enalapril 1.25 mg, nicorandil 15 mg, and tolvaptan 7.5 mg. One week after admission, he suddenly developed VF. An emergency cardiac catheterization was performed because of suspected ischemic involvement. For improvement of ischemia, an urgent percutaneous coronary intervention was performed for the occlusion of the left anterior descending coronary artery under intra-aortic balloon pump (IABP) support. Thus, he underwent successful revascularization. However, despite frequent defibrillation under deep sedation with amiodarone, VF developed into an electrical storm, and the patient was transferred to our hospital. Detailed blood sampling results from the previous hospital are presented in Table 1.

**Table 1:** Pre-hospital blood test results before being transferred to our hospital ALP = alkaline phosphatase; ALT = alanine aminotransferase; AST = aspartate aminotransferase; BUN = blood urea nitrogen; CPK = creatine phosphokinase; HCT = hematocrit; HGB = hemoglobin; LDH = lactate dehydrogenase; NT-proBNP = N-terminal-proBrain natriuretic peptide; PLT = platelet count; RBC = red blood cell count; WBC = white blood cell count

	Value	Normal range
NT-proBNP, pg/mL	3292	0-125
CPK, IU/L	96	56-244
CK-MB, IU/L	37	7.9-17.3
Cardiac troponin I, ng/mL	0.788	0-0.034
C-reactive protein, mg/dL	3.8	0-0.3
Glucose, mg/dL	180	80-200
Electrolytes		
Sodium (Na), mEq/mL	140.1	135-147
Potassium (K), mEq/mL	3.8	3.5-5.0
Creatinine, mg/dL	0.72	0.6-1.1
BUN, mg/dL	20.4	8-20
Complete blood cell count		
WBC, 10^2^/µL	88.1	35-80
RBC, 10^4^/µL	475	410-550
HGB, g/dL	13.7	13.4-17.4
HCT, %	39.6	39.8-51.5
PLT, 10^4^/µL	32.8	13-33
Total bilirubin, mg/dL	0.6	0.2-1.2
AST, IU/L	261	8-38
ALT, IU/L	248	4-44
ALP, IU/L	245	104-338
LDH, IU/L	747	106-211

His N-terminal-proBrain natriuretic peptide (BNP) was markedly elevated, but electrolytes were within normal limits, including potassium.

In our hospital, there was no evidence of acute stent thrombosis on coronary angiography (Figure 1B). After IABP was removed and emergent VA-ECMO (Cardiohelp HLS Set 7.0, 25 Fr venous cannula, 17 Fr arterial cannula; Maquet Cardiopulmonary, Hirrlingen, Germany) with assisted flow maintained at 3.3L/min, defibrillation was repeatedly performed 30 times; however, there was no sinus rhythm recovery (Figure 1C). Therefore, we tried terminating the electrical storm through left ventricular (LV) unloading by adjunctive Impella CP (Abiomed, Danvers, Massachusetts) with assisted flow maintained at 1.5 L/min and setting the Impella power level at P4 (Figure 1D). Consequently, the electrical storm was successfully terminated by the first external defibrillation just after Impella insertion; 23 minutes had passed since the last cardioversion (Figure 1E). Table 2 shows a comparison of blood sampling results and LV ejection fraction (EF) at pre-hospital, immediately after admission, and after resolved VF.

**Table 2:** Blood test results and left ventricular ejection fraction (LVEF) at three points

	Lactate (mg/dL)	Total bilirubin (mg/dL)	Creatinine (mg/dL)	Potassium (mEq/L)	LVEF (%)
Pre-hospital	15	0.6	0.72	3.8	34
Immediately after admission	10	0.8	0.67	3.7	No data
After resolved VF	20	1.0	0.71	4.2	36

**Figure 2:**
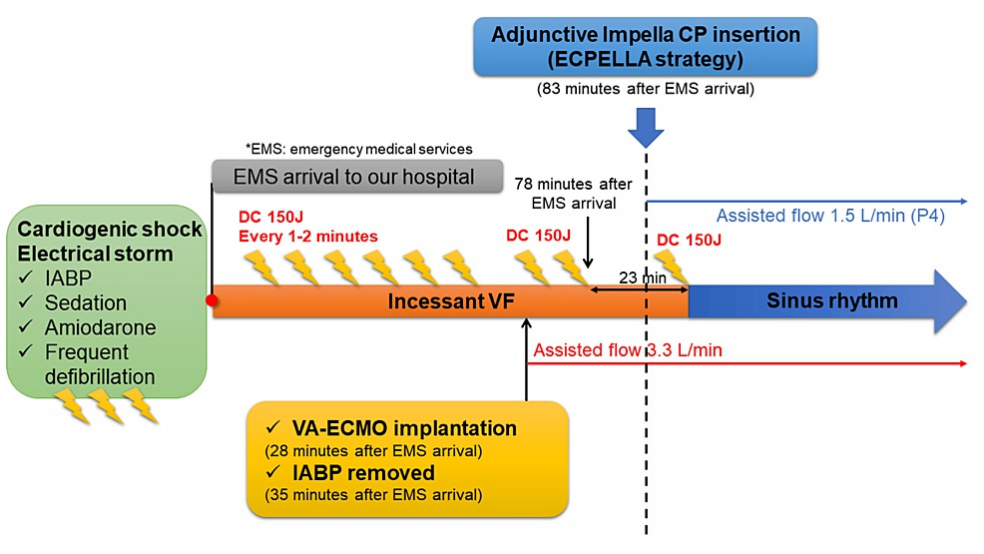
Clinical course after transfer to our hospital

Peak creatine phosphokinase (CPK) level was relatively low at 935 IU/L on day 3 of admission. In the present case, since time had passed since the myocardial infarction, it may not accurately reflect the peak CPK value. Under the mechanical circulatory supports, his hemodynamic state stabilized and gradually improved. LV end-diastolic diameter (LVDd) decreased from 59 mm to 51 mm 3 hours after Impella insertion. Moreover, the BNP level was 454 pg/mL on admission but decreased markedly to 227 pg/mL a few hours after admission to the intensive care unit (ICU) and 67 pg/mL two days later. Amiodarone was continued for a week, but lidocaine was not administered because the ventricular arrhythmia was under control. A summary of the clinical course is shown in Figure 2.

**Figure 3:**
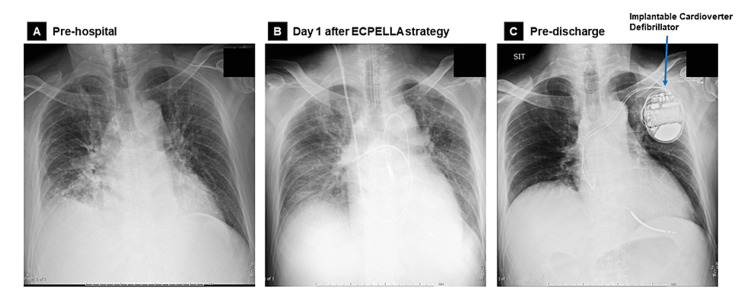
Progress of chest X-ray Chest X-ray at discharge (C) showing improvement in pulmonary congestion that had been observed in the previous hospital (A) and day 1 after insertion of ECPELLA (combined configuration of VA-ECMO and Impella) (B).

**Figure 4:**
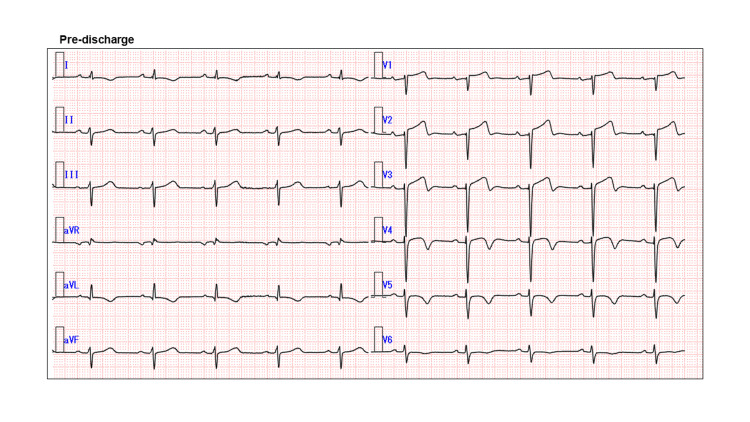
Electrocardiogram (ECG) at discharge ECG showing poor R progression in the precordial lead

**Figure 5:**
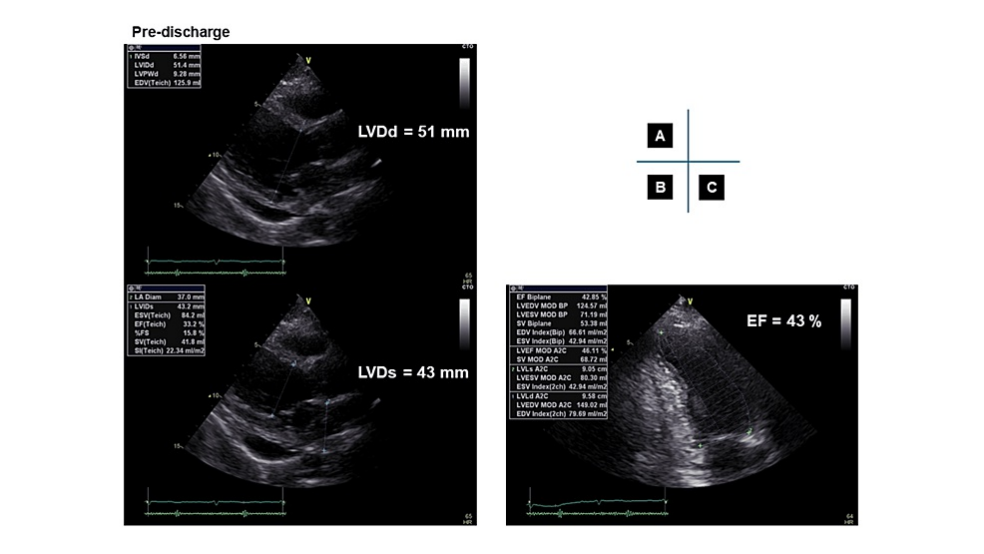
Echocardiographic evaluation of cardiac function at discharge Echocardiography showing left ventricular end-diastolic diameter (LVDd) of 51 mm (A) with 43 mm LV end-systolic diameter (LVDs) (B), and ejection fraction (EF) of 43% by the modified Simpson method (C)

First, the patient was weaned from VA-ECMO after seven days, then from Impella support after nine days with no recurrence of VF. The pulmonary artery pressure (PAP) and central venous pressure (CVP) at weaning from VA-ECMO and Impella were as follows, and no worsening was observed after weaning: (1) PAP of 30/16 mmHg, mean PAP of 21 mmHg, and CVP of 8 mmHg at weaning from VA-ECMO, (2) PAP of 25/15 mmHg, mean PAP of 20 mmHg, and CVP of 11 mmHg at weaning form Impella. Moreover, the lactate remained in the range of 5-8 mg/dL with no deterioration before or after weaning from mechanical circulatory supports; these results suggest successful weaning from mechanical assistance without exacerbation of circulatory failure. He was discharged without heart failure symptoms after an implantable cardioverter defibrillator implantation. On discharge from our hospital, the examination findings were as follows; (1) chest X-ray showed improvement of pulmonary congestion compared to chest X-rays at pre-hospital and one day after the ECPELLA strategy (combined configuration of VA-ECMO and Impella) (Figure 3), (2) 12-lead ECG showed sinus rhythm with HR 60/bpm and poor R progression in the precordial lead indicating anterior wall myocardial infarction (Figure 4), (3) echocardiography showed LVDd of 51 mm with a 43 mm end-systolic diameter. The EF was 43% by the modified Simpson method with abnormal wall motion from the mid-septum to the apex of the heart (Figure 5).

## Discussion

An electrical storm is a life-threatening condition that requires emergency management. However, mechanical support for cardiogenic shock secondary to refractory electrical storm is still challenging. The European Society of Cardiology (ESC) guidelines recommended that mechanical circulatory support may be considered in the management of drug-refractory electrical storms and cardiogenic shock [4]. VA-ECMO should be considered first to maintain systemic circulation during cardiac arrest or sustained VF. However, due to retrograde blood return, VA-ECMO increases the LV afterload and thus maintains a high LV end-diastolic pressure (EDP). Sustained loading of the heart due to pressure overload may prevent recovery from the electrical storm. Thus, further strategies might be considered for LV unloading when hemodynamic stabilization with VA-ECMO does not control VF. Lüsebrink E et al. reported that the additive insertion of IABP or Impella to VA-ECMO could be helpful in LV unloading in cardiogenetic shock [5]. However, it is still unclear what would be a better mechanical circulatory device to add to VA-ECMO.

The IABP is an option considered for the treatment of cardiogenic shock and has been used as an adjunct to VA-ECMO. However, since IABP is a balloon inflation device triggered by electrocardiogram or aortic pressure, it can be primarily effective in the setting of sinus rhythm, not VF [6]. On the other hand, Impella pumps blood from the LV into the ascending aorta even under VF and provides the desirable effect of lowering EDP when inserted in addition to VA-ECMO, called the ECPELLA strategy [7,8]. Schrage B et al. showed that adjunctive Impella insertion to VA-ECMO resulted in a marked decrease of pulmonary artery wedge pressure measured by right heart catheterization [7]. Moreover, Donker DW et al. demonstrated that the Impella was the most effective device to add to VA-ECMO for LV unloading in the analysis of LV pressure-volume loops [9]. Thus, adverse effects caused by VA-ECMO introduction could be ameliorated by LV unloading with Impella insertion. In fact, in this case, adjunctive Impella insertion resulted in hemodynamic stability, suggesting that Impella might be a useful mechanical adjunct to VA-ECMO during an electrical storm.

Patients with an electrical storm often preserve a hemodynamic state after VA-ECMO is introduced, but VF leads to progressive myocardial irritability due to inadequate coronary perfusion. Besides the effect of lowering EDP, Impella can be expected to increase coronary perfusion directly. Based on the above, we chose the strategy of inserting an Impella in addition to VA-ECMO for a patient in an electrical storm due to ischemic heart failure.

In this case, LVDd decreased immediately after the adjunctive Impella insertion. As a result, two hours after admission to the ICU, Impella’s flow was reduced from P4 to P2 due to the suction alarm sounding. Moreover, his BNP level was markedly decreased after the adjunctive Impella insertion. These results indicated successful LV unloading. Therefore, the adjunctive Impella insertion might have contributed to the recovery from electrical storm in the patient with ischemic heart failure by the following two mechanisms: (1) LV unloading, and (2) improvement of coronary circulation.

## Conclusions

In the present case, the electrical storm was not resolved despite performing defibrillation 30 times under VA-ECMO support. However, the refractory VF was terminated by the initial defibrillation after adjunctive Impella support and was completely controlled thereafter. This case illustrates the possible efficacy of adjunctive Impella insertion for a refractory electrical storm that is resistant to defibrillation under mechanical support with VA-ECMO for cardiogenic shock. Further validation is needed to verify the effectiveness.

## References

[1] Elsokkari I, Sapp JL (2021). Electrical storm: prognosis and management. Prog Cardiovasc Dis.

[2] Castelein T, Balthazar T, Adriaenssens T (2020). Impella to resist the storm. Circ Heart Fail.

[3] Martins RP, Maille B, Bessière F (2021). Left ventricular assist device implantation as a bailout strategy for the management of refractory electrical storm and cardiogenic shock. Circ Arrhythm Electrophysiol.

[4] Zeppenfeld K, Tfelt-Hansen J, de Riva M (2022). 2022 ESC Guidelines for the management of patients with ventricular arrhythmias and the prevention of sudden cardiac death. Eur Heart J.

[5] Lüsebrink E, Binzenhöfer L, Hering D (2024). Scrutinizing the role of venoarterial extracorporeal membrane oxygenation: has clinical practice outpaced the evidence?. Circulation.

[6] Jentzer JC, Noseworthy PA, Kashou AH (2023). Multidisciplinary critical care management of electrical storm: JACC state-of-the-art review. J Am Coll Cardiol.

[7] Schrage B, Burkhoff D, Rübsamen N (2018). Unloading of the left ventricle during venoarterial extracorporeal membrane oxygenation therapy in cardiogenic shock. JACC Heart Fail.

[8] Belohlavek J, Hunziker P, Donker DW (2021). Left ventricular unloading and the role of ECpella. Eur Heart J Suppl.

[9] Donker DW, Brodie D, Henriques JP, Broomé M (2019). Left ventricular unloading during veno-arterial ECMO: a review of percutaneous and surgical unloading interventions. Perfusion.

